# Zinc and low-dose of cadmium protect sertoli cells against toxic-dose of cadmium: The role of metallothionein

**Published:** 2013-06

**Authors:** Fatemeh Kheradmand, Issa Nourmohammadi, Mohamad Amin Ahmadi-Faghih, Mohsen Firoozrai, Mohammad Hossein Modarressi

**Affiliations:** 1 *Department of Biochemistry, Faculty of Medicine and Center for Cellular and Molecular Research, Urmia University of Medical Sciences, Urmia, Iran.*; 2 *Department of Biochemistry, Faculty of Medicine, Tehran University of Medical Sciences, Tehran, Iran.*; 3 *Department of Genetics, Faculty of Medicine, Tehran University of Medical Sciences, Tehran, Iran.*

**Keywords:** *Cadmium*, *Gene expression*, *Metallothionein*, *Sertoli cells*, *Zinc*

## Abstract

**Background:** The impact of cadmium (Cd) on male infertility may be related to the interaction with metal-binding proteins known as metallothioneins (Mts). Trace elements like zinc (Zn) have protective effects on testicular damage induced by Cd.

**Objective:** We determined the effect of Zn and low-dose Cd pre-treatment on the expression of *Mt1* and *Mt2* genes on testicular Sertoli cells.

**Materials and Methods:** The cultured TM4 mouse sertoli cells were treated with 50 μM ZnSO_4_ (Zn pre-treated group; ZnPG), 2 μM CdCl_2_ (Cd pre-treated group; CdPG), or distilled water (DW pre-treated group; DWPG). After 18 hour, all of these groups were exposed to 100 μM CdCl_2_ for different periods of time (1, 2, 3, and 6 hours). There was also a control group for all three groups, which was treated only with distilled water (without Cd or Zn pre-treatment). Cellular viability, Zn and Cd concentrations and gene expression were assessed by MTT, atomic absorption spectrometry and real time PCR methods, respectively.

**Results:** The expression of *Mt1* and *Mt2* genes in ZnPG, CdPG, and DWPG was greater than the control group (p=0.02 and p=0.01, respectively). Cd concentrations in CdPG and DWPG were greater than the control group (p=0.00). Expression of both genes in ZnPG and CdPG increased after 3 hours of treatment and Cd concentration decreased simultaneously, which was more obvious in ZnPG.

**Conclusion:** Zn and short term low-dose Cd pre-treatment might reduce the adverse effects of Cd by increasing expression of Mts genes in Sertoli cells. The protective effect of Zn was stronger than Cd.

## Introduction

The toxicity of cadmium (Cd) in the testis, can cause numerous disorders and diseases such as infertility and cancer. The testis is the target site of acute Cd toxicity. In rats, long-term administration of Cd in the diet was shown to induce pathological alterations in the testis with loss of reproductive capacity (1, 2). 

The role of Cd in the testis has been discussed in many studies, which have proposed several mechanisms for its effects, such as lipid peroxidation, alteration of copper (Cu) and zinc (Zn) homeostasis, oxidative stress and its effects on the hypothalamic-pituitary-testicular axis (3-8). Cd also induces changes in the testis, like disruption of tight-junctions in Sertoli cells, blood-testis barrier damage, testicular necrosis and imperfect spermatogenesis (9, 10). In addition, metallothioneins (Mts) as proteins, which are involved in resistance to Cd-induced tissue damage, are of great importance. These proteins can be induced by trace elements like Zn and Cd (3, 4, 11-13). The role of Mts as major proteins in protecting acute Cd poisoning came from the observation that pre-treatment of animals with a low dose of Cd renders animals highly tolerant to Cd-induced lethality in wild-type but not in Mts-null mice (14-16).

Jemai et al showed that pre-treatment with Zn exhibited a protective role against Cd toxicity with a significant decrease in serum Zn content because of excessive use of Zn in Mts synthesis as Cd detoxification agent (17). In recent years, it has been proposed that the development of certain reproductive disorders such as low sperm count and infertility, which may associate with Cd contamination, can be prevented by Zn (4, 18, 19). Zn is known to be particularly important in the development and function of sperm, and there are documented data that Zn supplementation improves sperm count, quality, and motility (20, 21). 

Zn and low-dose Cd have a protective effect on testicular damage induced by Cd (22-26). The mechanism of Zn protection against Cd toxicity is not precisely known yet, although some studies suggest an antioxidant role for Zn or Mts proteins (4, 27, 28). It has been also documented that Mts associate with Cd detoxification in the liver, kidney, and intestine. Little is known, however, about the role of Mts, especially regarding the preventive effect of Zn and low-dose Cd against Cd toxicity in testicular sertoli cells (25, 29-32). These cells are considered supportive cells in the seminiferous epithelium and appear to be highly sensitive to Cd cytotoxicity (3, 33). 

In the current study, we analyzed the effect of Zn and low-dose Cd pre-treatment on preventing later Cd toxicity by evaluating Mts genes expression in testicular Sertoli cells.

## Materials and methods

The methods of the study were reviewed and approved by institutional review board (IRB) of Iran University of Medical Sciences.

**Cell Culture**

TM4 mouse sertoli cells (ATCC number CRL-1715) were cultured as described by the ATCC protocol. Sertoli cells were grown in a mixture of Ham's F12 medium (Biosera, UK) and Dulbecco's Modified Eagle's Medium (Biosera, UK) supplemented with 1.2 g/L sodium bicarbonate, 15 mM HEPES [(4-(2-hydroxyethyl) -1 - piperazineethanesulfonic acid), (Sigma, USA)] plus 5% horse serum (Sigma, USA) and 5% fetal bovine serum (Sigma, USA). Cells were incubated at 37^o^C in a 5% humidified CO_2_-enriched atmosphere. Cd and Zn serial dilutions were prepared from a 10 mM stock CdCl_2_ and ZnSO_4_ solution, respectively. 

TM4 cells were pre-treated with 50 μM ZnSO4 (Zn pre-treated group; ZnPG) or 2 μM CdCl_2_ (Cd pre-treated group; CdPG) or distilled water (DW pre-treated group; DWPG). After 18 hours all of these groups were treated by a single dose of 100 μM CdCl_2_ for different periods of time (1, 2, 3, and 6 hours). There was also a control group for all three groups, which was treated only with distilled water (without Zn or Cd pre-treatment). Before any experiment, viable cell number was enumerated with trypan blue staining using a Neobar hemocytometer. There were four subgroups (1, 2, 3, and 6 hours) in each group and three replicates in all subgroups.

**Cell viability assay**

Cellular viability in the different groups was determined using MTT [3-(4, 5- dimethylthiazol-2-yl)-2, 5-dimethyl tetrazolium bromide assay] (Sigma, UK) in a final concentration of 0.5 mg/ml. Briefly, following treatments, 100 μl of culture medium containing MTT solution was added in each well (10×105 cells), and the plate was incubated for 2 hours at 37ºC. The medium was then removed and the colored reaction product was solubilized in 100 μl DMSO (Dimethyl sulfoxide). Absorbance was measured at 570 nm using an ELISA reader (Anthos 2020, Austria). The percentage viability was calculated as follows: Percentage specific viability= A/B×100, where A and B represent absorbance at 570 nm of the treated and untreated (control) samples. 

**Cd and Zn determination**

At the end of treatments (1, 2, 3, and 6 hours), both treated and untreated cells (10×10^5^ cells) were washed twice with cold phosphate buffered saline (PBS), and then total Zn and Cd concentrations were determined by flame atomic absorption spectrometry (FAAS) and graphite furnace atomic absorption spectrometry (GFAAS), respectively, using a Spectra AA 220 (Varian, Australia) spectrometer. To avoid Cd and Zn contamination, precautions were taken using acid washed glassware or plastic bottles for growth of cultures and deionized water to prepare the reagents. 

**RNA extraction**

For RNA extraction, the PBS washed treated cells (10×105 cells) were centrifuged twice for 5 min at 1600 g.Then total RNA was extracted from these cells using the High Pure RNAgents Isolation kit (Promega, UK). Briefly, the cell pellet was homogenized in 120 μl of prechilled denaturing solution from the system by vortexing for 5-10 min. The following reagents were added: 12 μl of 2 mol/L sodium acetate (pH=4.0), and 120 μl of phenol/chloroform/isoamyl alcohol mixture. The samples were vortexed thoroughly and chilled on ice for 20 min, then centrifuged at 10,000 g at 4oC for 20 min. 

The aqueous upper phase was transferred to a clean 1.5 ml microcentrifuge tube, and an equal volume of isopropanol was added. The tubes were vortexed and stored at -20oC for 20 min, then centrifuged at 10,000 g for 10 min at 4oC to precipitate RNA. The supernatant was removed and the RNA pellet was washed with 75% ethanol, centrifuged at 10,000 g for 10 min at 4oC, then vacuum dried, and resuspended in 20 μl of nuclease-free water and stored in aliquots at -80oC. Purity of RNA was estimated by the absorbance ratio A260/A280 nm. 

RNA integrity was confirmed by ethidium bromide staining of ribosomal RNA following gel electrophoresis. Synthesis of cDNA was performed according to a previous report using random hexamer and M-Mulv reverse transcriptase enzyme (Fermentas, Canada) (34). Two important isoforms of *Mts* (*Mt1* and *Mt2*) were selected for analysis of their expression in Sertoli cells. Primer design was done with regard to primer dimer formation, self-priming formation, and primer melting temperature, and was checked with Generunner software. Blast search in the published sequence database Gene Bank (http://blast.ncbi.nlm.nih.gov/Blast.cgi) revealed the primers to be gene specific (Table I). The beta actin gene was used as a housekeeping gene. 

**Quantitative real-time RT PCR analysis**

Real-time RT PCR was carried out with Quantifast SYBR Green Kit (Roche Applied Science, UK) using 1 μl of cDNA (equivalent to 100 ng cDNA) in a 20 μl final volume and 0.4 μM of each primer (final concentration). Quantitative PCR was performed using a Corbett Rotor-Gene 3000 for 35 cycles at 95oC for 10 seconds and at 60oC for 20 seconds. Specificity of product for each separate sample was checked with the melting curve analysis, and quantification was achieved with the comparative threshold cycle method.

**Statistical analysis**

This study was carried out as an experimental study. Statistical analysis was performed using the SPSS software (version 16.0). For data analysis, the independent t-test, Mann-Whitney-U-Test, one-way ANOVA analysis, and Kruskal-Wallis H test were performed. A p-value less than 0.05 was considered for significant difference. 

**Table I:** Sequences of the primers used in real-time PCR assays

**Gene name**		**Sequence**	**Product size (bp)**	**Accession number**
*Mt1*	F	5′-ATGGACCCCAACTGCTCCTG-3′	197	NM_013602.3
	R	5′-TTCGTCACATCAGGCACAGC-3′		
*Mt2*	F	5′-ACCCCAACTGCTCCTGTGCC-3′	127	NM_008630.2
	R	5′-CACTTCGCACAGCCCACGG-3′		
*Beta actin*	F	5′- GCTATGCTCTCCCTCACGCCA -3′	201	NM_007393.3
R	5′- AGGAAGAGGATGCGGCAGTGG-3′

*Mt1*: metallothionein-1;

*Mt2*: metallothionein-2;

F: forward;

R: reverse.

## Results

**The effect of treatments and pre-treatments on cellular viability**

**Figure 1:**
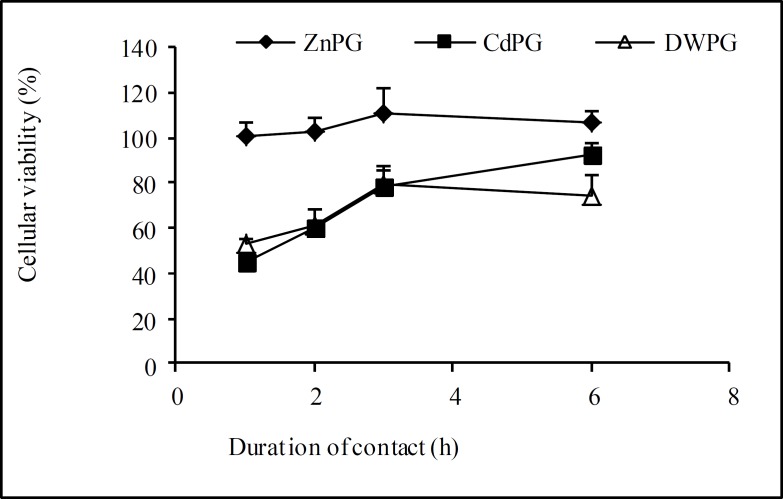
Cellular Viability in pre-treated Sertoli cells.

Figure 1 shows cellular viability at different times of treatments in the pre-treated cells. The cellular viability in ZnPG cells was near to that of the control group generally throughout the experiment. In two other groups (CdPG and DWPG) cellular viability decreased at first hour and then increased especially in CdPG cells (Figure 1, p=0.00).

**Determination of Cd and Zn concentration**

**Figure 2:**
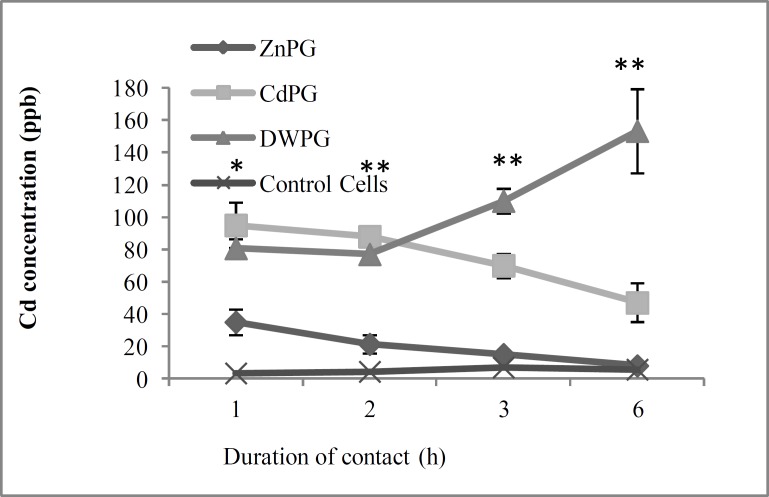
Cd concentration in pre-treated Sertoli cells and control group.

The Cd concentrations in CdPG and control group were 75.01±21.43 parts per billion (ppb) and 5.00±1.82 ppb, respectively (p=0.00). The differences in Cd concentration between ZnPG and the control group [(19.75±11.47 ppb vs. 5.00±1.82 ppm, (p=0.06)] and in Zn concentration [0.11±0.03 ppm in ZnPG vs. 0.12±0.03 ppm in the control group, (p=0.07)] were insignificant. In the cells pretreated with DW, Cd concentration was 105.25±35.06 ppb versus 5.00±1.82 ppb in the control group (p=0.00). According to figure 2, the Cd concentration in CdPG was lower than that in DWPG. In ZnPG, the Cd concentration was very low in comparison with two other groups (CdPG and DWPG). This figure also shows that Cd concentration in ZnPG and CdPG decreased up to 6 hours, whereas it increased in DWPG. 

**Mts genes expression**

**Figure 3:**
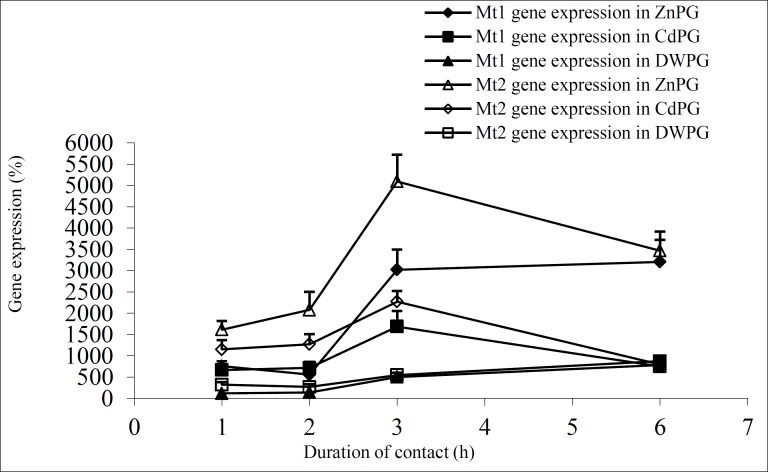
*Mts* genes expression in pre-treated Sertoli cells.

Table II shows the *Mts* genes expression in the three pretreated groups and the control group. According to this table, the expression of both* Mt1* and *Mt2* genes in all pretreated groups was greater than that in the control group (p=0.02 and p=0.00, respectively). Figure 3 represents the relative expression for *Mt1* and *Mt2* genes at different times of treatment. 

The expression of genes in DWPG compared with the two other groups (ZnPG and CdPG) was low, and Zn pre-treatment had a more prominent role in the excitement of gene expression.

**Table II:** *Mts* genes expression in three pretreated groups and control groups

	***Mt1*** ** gene expression**	**p value**	***Mt2*** ** gene expression**	**p value**
**CdPG**	1.9 ± 0.97	0.02	2.3 ± 1.03	0.00
**ZnPG**	3.77 ± 2.86		5.63 ± 3.43	
**DWPG**	1.24 ± 0.3		2.65 ± 1.3	
**CG**	0.42 ± 0.12	0.47 ± 0.23

CdPG: Cd pretreated group; ZnPG: Zn pretreated group; DWPG: DW pretreated group; CG: Control group. Data represent mean values ± SE of four subgroups (1, 2, 3, 6 hours) in each group and three replicates in all subgroups.

**Cellular viability in ZnPG cells was about the level of control cells. In the two other groups (CdPG and DWPG), cellular viability decreased to 45% for CdPG and 53% for DWPG at first hour of the test and then increased especially in CdPG cells. Data represent mean values ± S.E of six analyses (three replicates in all subgroups with two analyses per replicate). (p=0.00)**

Cd concentration in DWPG increased as time passed and reached 153 ppb in the sixth hour. In CdPG and ZnPG, Cd concentration decreased to 47 ppb and 8 ppb, respectively, in the sixth hour. In the control cells, the values were so low. Data represent mean values ± S.E of three replicates in all subgroups. Mean values were compared using one-way ANOVA. (*p = 0.01, **p = 0.00).

Expression of genes in DWPG in comparison with the two other groups was low (between 115% and 780%). In ZnPG, the expression of both genes was higher than in CdPG. The increase in gene expression was more obvious for the *Mt2* gene (5095% in the third hour and 3470% in the sixth hour) compared with the *Mt1* gene (3020% in the third hour and 3205% in the sixth hour). Data represent mean ± SE of three replicates in all subgroups.

## Discussion

Our results showed that pre-treatment with Zn and low-dose Cd caused an obvious increase in the expression of *Mt1* and *Mt2* genes in cultured Sertoli cells, which was more prominent during the last hours of the tests. Besides, decreasing Cd concentration in ZnPG and CdPG cells showed the efficiency of pre-treatment in preventing Cd toxicity over time. All of the above considerations, as well as the closeness of cellular viability to the control group in ZnPG cells compared with the two other groups, highlighted the role of Zn as a toxicity-preventing factor more than low-dose Cd. It seems that in ZnPG, increasing Mts proteins might decrease Cd content to normal level.

We also found that low-dose Cd increased Mts genes expression, which might cause cellular defence against toxic dose of Cd. Consistent with our results, some studies have indicated that the short term low-dose Cd may resist against subsequent toxicity of high-dose Cd by increasing the expression of Mts genes in animals, isolated interstitial cells, and Sertoli cells (14-16, 24, 35). In contrast Miura et al have reported that Cd exposure can lead to long-term effects on human health even at low concentrations (36). 

Zhang *et al* have found that CdCl_2_ can induce apoptosis of Sertoli cells even at a low concentration of 10 μM (37). Furthermore Panjehpour *et al* have indicated that low-dose Cd was very cytotoxic in the human lung carcinoma cell line. As Mts induction is dose- and time-dependent in Sertoli cells, therefore, some of the controversy may derive from the different study time and used dose (38). 

Kusakabe *et al* also have reported that Cd-induced Mts proteins protect Sertoli cells against apoptosis (3); but, Ren *et al* in their research have shown that Cd exposure, despite the increase in cellular *Mts* genes expression, do not increase Mts proteins levels in Sertoli and spermatogenic cells. They have demonstrated that the inability to induce the metal-detoxicating Mts proteins may account for higher susceptibility of testis to Cd toxicity (45, 46). Unfortunately, we did not investigate the levels of Mts proteins in our study.

About the effect of Zn, similar to our study, Hu et al. have shown positive effect of Zn treatment on the *Mt2* gene in prostate and testis of rats (26). Wahba *et al* have mentioned opposite outcomes in their research, which might be due to the use of different methods in analyzing gene expression (39). However, to the best of our knowledge, there has been so far no report about the protective function of Zn pre-treatment against Cd toxicity especially in Sertoli cells. However, some data have indicated the protective effects of Zn-induced Mts against Cd toxicity in the liver and kidney (40-43). 

Our results showed that Mts have a prominent role in the protective effect of Zn against Cd toxicity in these cells. Cd binds to Mts more tightly than does Zn (44). In high Cd status the cell encounters two problems: Cd toxicity and concomitant Zn deficiency, which arises from Cd excess as mentioned by Bonda *et al* (40). Therefore, increase of Mts concentration by Zn pre-treatment can increase the cellular Zn content and remove toxic Cd. 

Obviously, more studies should be undertaken to investigate the proteins level of Mts and on other cell types of the testis. In addition, evaluating morphologic and histological changes can be helpful to assess the effect of Zn and Cd pre-treatments in the gross index of cells. Moreover, examining other Zn dosages, especially on longer periods, may provide better explaining of the protection mechanism of Zn.
